# RHYTHM AND NATURE: APPLYING TRAUMA-INFORMED APPROACHES TO RESEARCH TO PRIORITIZE CHOICE, EXPRESSION, AND JUSTICE

**DOI:** 10.1093/geroni/igad104.2351

**Published:** 2023-12-21

**Authors:** Emily Ihara, Michelle Hand

**Affiliations:** George Mason University, Fairfax, Virginia, United States; George Mason University, Fairfax, Virginia, United States

## Abstract

Trauma-informed approaches are widespread in direct clinical and particularly in social work practice, and have gained momentum in other areas such as research, organizational culture, supervision, and leadership. Given the recent collective global trauma of the pandemic and the cumulative effects of historic and ongoing racial injustice, a trauma-informed approach to research with older adults, especially with those who have disabilities, such as relating to dementia, can contribute to the movement for access, justice, equity, and inclusion for all. Closely aligned with practices of community-based participatory action research, trauma-informed research pays particular attention to the consideration of the design of studies, the analytical and theoretical frameworks, collaboration among researchers and participants, data collection methods, research questions that capture the knowledge and experiences of participants’ resiliency, strengths, and challenges, and the processes of reflexivity. Rhythm & Nature was a 10-week pilot study designed to incorporate trauma-informed research approaches to promote principles of safety, trustworthiness, collaboration, choice, self-expression, and justice. We created a modifiable drumming and nature-based intervention to promote opportunities for self-expression and confidence. Before and after each drumming and nature-based session, we collected data using emojis (e.g., to increase access for people with limited language or literacy) to indicate how participants felt in the moment and used their ongoing feedback to continuously tailor the program to their unique abilities and needs. Both quantitative and qualitative results of this pilot study and implications for future research, policy, practice and education with older adults, particularly with disabilities, will be discussed.

